# Supplementary material for: Comparison of Antibiofilm Activity of Pseudomonas aeruginosa Phages on Isolates from Wounds of Diabetic and Non-Diabetic Patients
Source: Microorganisms. 2023 Sep 4;11(9):2230. doi: 10.3390/microorganisms11092230 (PMC10536433; doi:10.3390/microorganisms11092230)
Supplement: Supplementary file 1 [file microorganisms-11-02230-s001.zip › microorganisms-2463936-supplementary.pdf]

**Table S1: Antimicrobial susceptibility of the bacterial isolates used in this study.** Represented as zone of inhibition in mm (R/S/I). R : Resistant ; I :Intermediate : S Susceptible.

| **SOURCE OF ISOLATES** | **ISOLATES** | **ANTIBIOTICS** | | | | | | | | | | | | |
|---|---|---|---|---|---|---|---|---|---|---|---|---|---|---|
| | | **Aminoglycosides** | | | | **Cephalosporins** | | | **Cabapenem** | **Betalactum with inhibitor** | | **Fluoroquinolones** | | **Monobactum** |
| | | **Gen** | **TOB** | **AK** | **NET** | **CPZ** | CAZ | **CFS** | **IPM** | **PI** | **PIT** | **LE** | **CIP** | **AT** |
| Standard culture | ATCC 27853 | 24 (S) | 32(S) | 18(S) | 26(S) | 29 (S) | 27(S) | 24(S) | 27(S) | 23 (S) | 27(S) | 31(S) | 20(S) | 27(S) |
| Diabetic wounds | ADWS6 | R | 15( R) | R | 22(S) | 22(S) | 25(S) | 21(I) | 28(S) | 16(I) | 25(S) | R | R | 28(S) |
| | ADWS 9 | R | 10(R) | 10(R) | 20(S) | 21(S) | 20(I) | 17(I) | 25(S) | 17(I) | 22(S) | R | R | 25(S) |
| | ADWS 10 | 9(R) | 20(S) | 16(I) | R | R | 14(R) | R | 15(R) | R | 16(I) | 21(S) | 15(S) | 15(R) |
| | ADWS 11 | R | 20(I) | R | R | R | R | R | R | R | R | 16(I) | R | R |
| | ADWS 14 | 13(I) | 30(S) | R | 20(S) | 20(I) | 22(S) | 21(I) | 26(S) | R | 21(S) | 19(S) | 23(S) | 26(S) |
| | ADWS 17 | 21(S) | 35(S) | 20(S) | 13(R) | R | 15(I) | 13(R) | 24(S) | R | 20(I) | 29(S) | 23(S) | 24(S) |
| | ADWS 23 | 22(S) | 33(S) | 17(S) | 20(S) | 16(I) | 19(I) | 15(R) | 21(S) | 16(I) | 21(S) | 29(S) | 23(S) | 21(S) |
| | ADWS 28 | 23(S) | 28(S) | 21(S) | 25(S) | 26(S) | 26(S) | 24(S) | 23(S) | 25(S) | 23(S) | 34(S) | 26(S) | 23(S) |
| | ADWS 36 | R | 32(S) | R | 33(S) | 24(S) | 21(S) | 30(S) | 15(R) | 28(S) | 26(S) | R | 32(S) | 15(R) |
| | ADWS 44 | R | 17(I) | 23(S) | 28(S) | R | 28(S) | R | 27(S) | R | 20(I) | R | 28(S) | 27(S) |
| | ADWS 46 | 30(S) | 11(R) | 25(S) | 20(S) | R | 27(S) | 21(I) | 27(S) | 24(S) | 31(S) | 25(S) | 24(S) | 27(S) |
| | ADWS 47 | 25(S) | 13(R) | 20(S) | 10(R) | 11(R) | 20(I) | 14(R) | 20(S) | R | 24(S) | 21(S) | 20(S) | 20(S) |
| | ADWS 55 | 20(S) | 40(S) | 23(S) | 24(S) | 20(I) | 22(S) | 24(S) | 21(S) | 26(S) | 24(S) | 27(S) | 22(S) | 21(S) |
| | ADWS 58 | 12(R) | 21(S) | 18(S) | 21(S) | 25(S) | 23(S) | 14(R) | 23(S) | 22(S) | 31(S) | R | 22(S) | 23(S) |
| | ADWS 62 | 25(S) | 31(S) | 24(S) | 24(S) | 25(S) | 24(S) | 20(I) | 24(S) | 27(S) | 21(S) | 22(S) | 21(S) | 24(S) |
| | ADWS 66 | 21(S) | R | 25(S) | 19(S) | R | 28(S) | 20(I) | 28(S) | 21(S) | R | R | 23(S) | 28(S) |
| | | | | | | | | | | | | | | |
| Non-diabetic wounds | ADWS 4 | 18(S) | 35(S) | 18(S) | 26(S) | 25(S) | 25(S) | 25(S) | 30(S) | 21(S) | 26(S) | 30(S) | 22(S) | 30(S) |
| | ADWS 8 | 23(S) | 33(S) | 22(S) | 18(S) | 23(S) | 17(I) | 16(I) | 22(S) | 20(I) | 17(I) | 35(S) | 24(S) | 22(S) |
| | ADWS 13 | 22(S) | 31(S) | 20(S) | 22(S) | 29(S) | 20(I) | 16(I) | 25(S) | 20(I) | 21(S) | 30(S) | 21(S) | 25(S) |
| | ADWS 15 | 21(S) | 40(S) | 22(S) | 25(S) | 20(I) | 17(I) | 17(I) | 24(S) | 15(I) | 19(I) | 32(S) | 22(S) | 24(S) |
| | ADWS 16 | 14(I) | 17(I) | 10(R) | R | R | 10(R) | R | 8(R) | R | 10(R) | R | R | 8(R) |
| | ADWS 21 | 21(S) | 26(S) | 15(I) | 22(S) | 18(I) | 25(S) | 21(I) | 21(S) | 20(I) | 23(S) | 30(S) | 22(S) | 21(S) |

| | | | | | | | | | | | | | | |
|---|---|---|---|---|---|---|---|---|---|---|---|---|---|---|
| | ADWS 25 | 26(S) | 32(S) | 10(R) | R | R | 11(R) | R | R | R | R | 21(S) | 31(S) | R |
| | ADWS 30 | 25(S) | 26(S) | 20(S) | 15(I) | 21(S) | 25(S) | 24(S) | 15(R) | 30(S) | 27(S) | 35(S) | 16(S) | 15(R) |
| | ADWS 34 | 25(S) | 24(S) | 26(S) | 21(S) | 23(S) | 22(S) | 15(R) | 30(S) | 25(S) | 25(S) | 36(S) | 21(S) | 30(S) |
| | ADWS 38 | 20(S) | 36(S) | 22(S) | 30(S) | R | 26(S) | 16(I) | 24(S) | 32(S) | R | 25(S) | 20(S) | 24(S) |
| | ADWS 40 | 11(R) | 10(R) | 10(R) | R | 28(S) | 12(R) | 17(I) | 7(R) | 25(S) | 16(I) | R | R | 7(R) |
| | ADWS 41 | 16(S) | 11(R) | 14(R) | R | 30(S) | 30(S) | 19(I) | 11(R) | 28(S) | R | R | R | 11(R) |
| | ADWS 43 | 25(S) | 32(S) | 21(S) | R | 31(S) | 29(S) | 16(I) | 24(S) | 38(S) | 28(S) | 26(S) | 21(S) | 24(S) |
| | ADWS 50 | 18(S) | 22(S) | 18(S) | 20(S) | 24(S) | 28(S) | 21(I) | 21(S) | 22(S) | 25(S) | 23(S) | 18(S) | 21(S) |
| | ADWS 51 | 27(S) | 25(S) | 22(S) | 20(S) | 24(S) | 30(S) | 15(R) | 30(S) | 21(S) | R | 24(S) | 22(S) | 30(S) |
| | ADWS 52 | 10(R) | 18(I) | 18(S) | 25(S) | 17(I) | 11(R) | R | 12 ( R) | 22(S) | 16(I) | R | 23(S) | 22(S) |
| | ADWS 53 | 31(S) | R | 15(I) | R | 23(S) | 10(R) | R | 11(R) | 21(S) | 8(R) | 15(I) | 16(S) | 11(R) |
| | ADWS 54 | 32(S) | 12(R) | 15(I) | 26(S) | 18(I) | 22(S) | 10(R) | 10(R) | R | R | 15(I) | 15(S) | 10(R) |
| | ADWS 56 | 26(S) | 37(S) | 23(S) | 18(S) | 21(S) | 13(R) | 21(I) | 26(S) | 26(S) | 26(S) | 24(S) | 23(S) | 26(S) |
| | | | | | | | | | | | | | | |
| | AMO1 | 24(S) | 32(S) | 21(S) | 28(S) | 32 (S) | 27(S) | 25(S) | 27(S) | 26 (S) | 27(S) | 33(S) | 23(S) | 28(S) |
| | JMO2 | 24(S) | 32(S) | 21(S) | 28(S) | 28 (S) | 27(S) | 25(S) | 27(S) | 26 (S) | 27(S) | 33(S) | 23(S) | 28(S) |
| | VMO2 | 24(S) | 32(S) | 21(S) | 28(S) | 27 (S) | 27(S) | 25(S) | 27(S) | 26 (S) | 27(S) | 33(S) | 23(S) | 28(S) |
| | VMO1 | 24(S) | 32(S) | 21(S) | 28(S) | 31 (S) | 27(S) | 25(S) | 27(S) | 26 (S) | 27(S) | 33(S) | 23(S) | 28(S) |
| | P-a1 | 24(S) | 32(S) | 21(S) | 28(S) | 30 (S) | 27(S) | 25(S) | 27(S) | 26 (S) | 27(S) | 33(S) | 23(S) | 28(S) |
| | JPA1 | 24(S) | 32(S) | 21(S) | 28(S) | 32 (S) | 27(S) | 25(S) | 27(S) | 26 (S) | 27(S) | 33(S) | 23(S) | 28(S) |
| | FTOA | 26(S) | 22(S) | 23(S) | 20(S) | 21(S) | 28(S) | 25(S) | 26(S) | 26(S) | 25(S) | 24(S) | 18(S) | 26(S) |
| Environment | FO1 | 24(S) | 32(S) | 21(S) | 28(S) | 32 (S) | 27(S) | 25(S) | 27(S) | 26 (S) | 27(S) | 33(S) | 23(S) | 28(S) |
| | VAO1A | 24(S) | 32(S) | 21(S) | 28(S) | 28 (S) | 27(S) | 25(S) | 27(S) | 26 (S) | 27(S) | 33(S) | 23(S) | 28(S) |
| | NOA | 24(S) | 32(S) | 21(S) | 28(S) | 32 (S) | 27(S) | 25(S) | 27(S) | 26 (S) | 27(S) | 33(S) | 23(S) | 28(S) |
| | VAO1B | 24(S) | 32(S) | 21(S) | 28(S) | 29(S) | 27(S) | 25(S) | 27(S) | 26 (S) | 27(S) | 33(S) | 23(S) | 28(S) |
| | FTOC | 24(S) | 32(S) | 21(S) | 28(S) | 32 (S) | 27(S) | 25(S) | 27(S) | 26 (S) | 27(S) | 33(S) | 23(S) | 28(S) |
| | FTO1a | 24(S) | 32(S) | 21(S) | 28(S) | 32 (S) | 27(S) | 25(S) | 27(S) | 26 (S) | 27(S) | 33(S) | 23(S) | 28(S) |
| | FLO1 | 24(S) | 32(S) | 21(S) | 28(S) | 32 (S) | 27(S) | 25(S) | 27(S) | 26 (S) | 27(S) | 33(S) | 23(S) | 28(S) |
| | FLO2 | 24(S) | 32(S) | 21(S) | 28(S) | 32 (S) | 27(S) | 25(S) | 27(S) | 26 (S) | 27(S) | 33(S) | 23(S) | 28(S) |
| | FO2 | 24(S) | 32(S) | 21(S) | 28(S) | 32 (S) | 27(S) | 25(S) | 27(S) | 26 (S) | 27(S) | 33(S) | 23(S) | 28(S) |

| | | | | | | | | | | | | | | |
|---|---|---|---|---|---|---|---|---|---|---|---|---|---|---|
| | SO1 | 24(S) | 34(S) | 21(S) | 24(S) | 32 (S) | 22(S) | 24(S) | 27(S) | 26 (S) | 24(S) | 33(S) | 22(S) | 28(S) |
| | SO2 | 24(S) | 32(S) | 21(S) | 28(S) | 32 (S) | 27(S) | 25(S) | 27(S) | 26 (S) | 27(S) | 33(S) | 23(S) | 28(S) |
| | JPO1 | 24(S) | 32(S) | 21(S) | 28(S) | 32 (S) | 27(S) | 25(S) | 27(S) | 26 (S) | 27(S) | 33(S) | 23(S) | 28(S) |

**Table S2: Antibiofilm activity of the phages on isolates from different sources.** Values displayed in mean absorbance at 570nm ± standard deviation

| | | Control | Pa1 | Pa3 | Pa6 | Pa7 | Pa9 | Pa10 | Pa14 | Pa15 | Pa18 |
|---|---|---|---|---|---|---|---|---|---|---|---|
| PAO1 | | 0.494 ±0.023 | 0.232 ± 0.094 | 0.278 ± 0.075 | 0.328 ± 0.059 | 0.271 ± 0.051 | 0.158 ± 0.008 | 0.215 ±0.008 | 0.164 ± 0.006 | 0.257 ± 0.008 | 0.256 ±0.019 |
| Non-diabetic wound isolates | ADWS04 | 1.134± 0.009 | 0.346 ± 0.067 | 0.208± 0.069 | 0.605 ± 0.025 | 0.259± 0.068 | 0.191± 0.006 | 0.190 ± 0.006 | 0.158 ± 0.013 | 0.286 ± 0.006 | 0.157± 0.007 |
| | ADWS08 | 0.942 ± 0.113 | 0.346 ± 0.081 | 0.857 ± 0.102 | 0.391 ± 0.069 | 0.482 ± 0.056 | 0.774 ± 0.039 | 0.548 ± 0.040 | 0.424 ± 0.008 | 0.301 ± 0.035 | 0.379 ±0.014 |
| | ADWS13 | 0.512 ± 0.071 | 0.257 ± 0.028 | 0.182 ± 0.020 | 0.152 ± 0.011 | 0.208 ± 0.022 | R | 0.146 ± 0.004 | 0.177 ± 0.007 | 0.174 ± 0.006 | 0.156 ± 0.004 |
| | ADWS15 | 1.464 ± 0.213 | 0.366 ± 0.068 | 0.287 ± 0.065 | 0.323 ± 0.044 | 0.219 ± 0.021 | 0.844 ± 0.043 | 0.184 ± 0.007 | 0.137 ± 0.008 | 0.299 ±0.008 | 0.354 ± 0.039 |
| | ADWS16 | 0.929 ± 0.037 | 0.829 ± 0.084 | 0.279 ± 0.026 | 0.647 ± 0.077 | 0.663 ± 0.047 | 0.38 ±0.005 | 0.407 ± 0.005 | 0.4 ± 0.046 | 0.49 ± 0.047 | R |
| | ADWS21 | 1.428 ± 0.064 | 0.924 ± 0.060 | 0.243 ± 0.049 | 0.144 ± 0.025 | R | 0.282 ± 0.026 | 0.159 ± 0.017 | 0.216 ± 0.008 | 0.195 ± 0.007 | 0.231 ± 0.008 |
| | ADWS25 | 0.716 ± 0.051 | 0.634 ± 0.027 | 0.481 ± 0.037 | 0.339 ± 0.031 | R | 0.479 ± 0.016 | 0.386 ± 0.008 | 0.447 ± 0.023 | 0.258 ± 0.023 | R |
| | ADWS30 | 1.021± 0.080 | R | 0.484 ± 0.071 | 0.339 ± 0.070 | 0.545 ± 0.047 | R | 0.375 ± 0.009 | R | 0.382 ± 0.04 | 0.343 ± 0.007 |
| | ADWS34 | 0.459 ± 0.059 | 0.404 ± 0.057 | 0.385 ± 0.034 | 0.286 ± 0.030 | 0.349 ± 0.030 | 0.369 ± 0.006 | 0.402 ± 0.048 | 0.33 ± 0.007 | 0.346 ± 0.036 | 0.386 ± 0.017 |
| | ADWS38 | 0.451 ± 0.036 | 0.216 ± 0.074 | 0.075 ± 0.003 | 0.057 ±0.003 | 0.072 ± 005 | 0.143 ± 0.009 | 0.081 ± 0.009 | 0.254 ± 0.011 | 0.161 ± 0.021 | 0.078 ± 0.011 |
| | ADWS40 | 1.072 ± 0.168 | 0.525 ± 0.053 | 0.483 ± 0.031 | 0.448 ±0.044 | 0.336 ± 0.018 | 1.029 ± 0.115 | 0.571 ± 0.049 | 0.456 ± 0.009 | 0.445 ± 0.041 | 0.448 ± 0.007 |
| | ADWS41 | 0.940 ± 0.049 | 0.192 ± 0.060 | 0.083 ± 0.003 | 0.557 ± 0.007 | 0.526 ± 0.065 | 0.086 ± 0.011 | 0.298 ± 0.015 | 0.312 ± 0.005 | 0.345 ± 0.017 | 0.624 ± 0.008 |
| | ADWS43 | 0.782 ± 0.041 | R | 0.605 ± 0.073 | R | 0.395 ± 0.057 | 0.483 ± 0.051 | 0.306 ± 0.032 | 0.369 ± 0.008 | 0.328 ± 0.009 | 0.282 ± 0.013 |
| | ADWS50 | 1.109 ± 0.05 | 0.415 ± 0.043 | 0.358 ± 0.032 | 0.633 ± 0.008 | 0.331 ± 0.007 | 0.358 ± 0.006 | 0.551 ± 0.026 | 0.349 ± 0.006 | 0.392 ± 0.016 | 0.581 ± 0.030 |
| | ADWS51 | 0.353± 0.015 | R | 0.239 ± 0.008 | 0.166 ± 0.003 | 0.185 ± 0.011 | 0.183 ± 0.015 | 0.185 ± 0.008 | 0.196 ± 0.006 | 0.198 ± 0.007 | 0.18025 |
| | ADWS53 | 0.743 ± 0.041 | R | 0.694 ± 0.057 | 0.281 ± 0.009 | 0.225 ± 0.025 | 0.549 ± 0.042 | 0.355 ± 0.04 | 0.272 ± 0.028 | 0.253 ± 0.028 | 0.232 ± 0.021 |
| | ADWS54 | 0.765 ± 0.047 | 0.699 ± 0.033 | R | R | 0.569 ± 0.012 | R | 0.501 ± 0.070 | 0.533 ± 0.037 | 0.511 ± 0.048 | 0.667 ± 0.015 |
| | ADWS56 | 1.169 ± 0.057 | 0.877 ± 0.068 | 0.530 ± 0.066 | 0.261 ± 0.017 | 0.332 ± 0.07 | 0.267 ± 0.031 | 0.370 ± 0.017 | 0.346 ± 0.018 | 0.337 ± 0.009 | 0.339 ± 0.037 |
| Diabetic wound isolates | ADWS06 | 0.911 ± 0.046 | 0.307 ±0.011 | 0.261 ± 0.007 | 0.272 ±0.006 | 0.251 ± 0.006 | 0.218 ± 0.006 | 0.219 ± 0.007 | 0.265 ± 0.019 | 0.253 ± 0.025 | 0.229 ± 0.017 |
| | ADWS09 | 0.884 ± 0.018 | R | 0.357± 0.036 | R | R | R | R | 0.273 ± 0.035 | R | R |
| | ADWS10 | 0.69 ± 0.031 | 0.606 ± 0.061 | 0.604 ± 0.067 | R | R | R | 0.412 ± 0.033 | 0.306 ± 0.036 | 0.196 ± 0.006 | R |
| | ADSW11 | 0.616 ± 0.045 | 0.153 ± 0.005 | 0.027 ± 0.002 | 0.358 ± 0.034 | R | 0.147 ± 0.007 | 0.174 ± 0.012 | 0.083 ± 0.011 | 0.117 ± 0.013 | 0.374 ± 0.016 |
| | ADWS14 | 0.778 ± 0.050 | 0.510 ± 019 | 0.371 ± 0.007 | 0.267± 0.007 | 0.382 ± 0.032 | 0.177 ± 0.016 | 0.179 ± 0.004 | 0.173 ± 0.004 | 0.213 ± 0.029 | R |
| | ADWS17 | 1.113 ± 0.109 | 0.156 ± 0.009 | 0.257± 0.011 | 0.164 ± 0.014 | 0.312 ± 0.007 | 0.238 ±0.011 | 0.296 ± 0.006 | 0.194 ± 0.006 | 0.235 ± 0.014 | 0.206 ± 0.027 |
| | ADWS23 | 0.937 ± 0.025 | 0.479 ± 0.054 | 0.199 ± 0.009 | 0.243 ± 0.005 | 0.208 ± 0.024 | 0.192 ± 0.007 | 0.164 ± 0.013 | 0.188 ± 0.023 | 0.244 ± 0.023 | 0.212 ± 0.004 |

| | | | | | | | | | | | |
|---|---|---|---|---|---|---|---|---|---|---|---|
| | ADWS28 | 0.597 ± 0.037 | 0.500 ± 0.028 | 0.488 ± 0.028 | R | R | 0.309 ± 0.016 | 0.362 ±0.007 | R | R | R |
| | ADWS36 | 0.86 ± 0.015 | R | R | 0.461 ± 0.02 | R | R | 0.366 ± 0.007 | 0.34 ± 0.033 | 0.359 ± 0.040 | R |
| | ADWS46 | 0.569 ± 0.011 | 0.488 ± 0.041 | 0.457 ± 0.041 | R | 0.516± 0.063 | 0.496 ± 0.025 | 0.566 ± 0.05 | 0.477 ± 0.008 | 0.504 ± 0.006 | 0.471 ±0.044 |
| | ADWS62 | 0.625 ± 0.061 | 0.332 ± 0.009 | 0.371 ± 0.029 | R | R | 0.308 ± 0.032 | R | 0.358 ± 0.020 | 0.277 ± 0.008 | 0.211 ±0.019 |
| | ADWS66 | 0.634 ± 0.017 | 0.181± 0.004 | 0.147± 0.004 | R | R | 0.289 ± 0.005 | 0.338 ± 0.009 | 0.301 ± 0.008 | R | 0.307 ± 0.018 |
| Environmental Isolates | AMO1 | 0.471 ± 0.034 | 0.534± 0.027 | 0.286 ± 0.035 | 0.451± 0.034 | 0.255 ± 0.014 | 0.059 ± 0.003 | 0.054± 0.009 | 0.172 ± 0.011 | 0.492 ± 0.022 | 0.242 ± 0.013 |
| | JMO2 | 1.839 ± 0.042 | 0.472± 0.023 | 0.198± 0.025 | 0.174± 0.011 | 0.149± 0.022 | 0.112± 0.016 | 0.1 ± 0.002 | 0.104± 0.015 | 0.227± 0.041 | 0.185 ± 0.033 |
| | VMO2 | 0.681 0.053 | 0.115 ± 0.012 | 0.335 ± 0.027 | 0.110 ± 0.019 | 0.278± 0.019 | 0.143± 0.011 | 0.120± 0.009 | 0.291± 0.021 | 0.137 ± 0.008 | 0.225 ± 0.046 |
| | VMO1 | 1.059 ± 0.056 | 0.343± 0.049 | 0.186± 0.007 | 0.098± 0.007 | 0.161± 0.022 | 0.128± 0.005 | 0.109 ± 0.010 | 0.364± 0.030 | 0.268± 0.005 | 0.156± 0.025 |
| | P-a1 | 1.249 ± 0.014 | 0.089 ± 0.009 | 0.251 ± 0.045 | 0.111 ± 0.009 | R | 0.159 ± 0.018 | R | R | 0.125 ± 0.013 | 0.209 ± 0.028 |
| | FTOA | 1.529 ± 0.039 | 0.1 ± 0.012 | 0.143± 0.016 | 0.164 ± 0.008 | R | 0.189 ± 0.013 | R | R | 0.163 ± 0.012 | 0.163 ± 0.018 |
| | FO1 | 1.348 ± 0.039 | 0.117 ± 0.010 | 0.202 ± 0.014 | 0.224 ± 0.010 | R | 0.184 ± 0.028 | R | R | 0.365 ± 0.036 | 0.220 ± 0.027 |
| | VAO1A | 0.946 ± 0.039 | R | 0.152±0.021 | R | R | 0.141±0.017 | R | R | 0.153± 0.016 | R |
| | NOA | 1.277± 0.048 | 0.231± 0.004 | 0.249± 0.096 | R | R | R | R | R | 0.148±0.017 | 0.167±0.017 |
| | VAO1B | 0.942± 0.017 | R | R | R | R | 0.174±0.011 | 0.349±0.020 | R | R | 0.206± 0.027 |
| | FTOC | 1.544 ± 0.055 | R | 0.271± 0.019 | R | 0.154± 0.014 | 0.154± 0.015 | R | R | R | 0.168± 0.012 |
| | FTO1a | 1.303 ± 0.031 | 0.094± 0.008 | 0.270± 0.023 | R | 0.387± 0.022 | 0.147± 0.015 | R | R | 0.164± 0.005 | 0.198± 0.013 |
| | FLO1 | 1.952 ± 0.05 | R | R | R | R | R | R | R | 0.009 ± 0003 | 0.018 ± 0.005 |
| | FLO2 | 0.449 ± 0.046 | R | 0.047± .0013 | R | 0.063 ± 0.007 | R | R | R | R | 0.034±0.008 |
| | FO2 | 2.241± 0.030 | R | 0.026 ± 0.006 | R | 0.012 ± 0.007 | R | 0.015± 0.008 | 0.023±0.004 | R | 0.058±0.014 |
| | SO1 | 1.644 ± 0.028 | 0.118± 0.006 | 0.122±0.014 | R | 0.094±0.014 | R | R | R | R | R |
| | SO2 | 0.510± 0.006 | R | 0.069±0.006 | R | 0.048± 0.005 | R | R | R | R | R |
| | JPO1 | 1.581± 0.036 | R | 1.101± 0.052 | R | 0.310± 0.012 | 0.184± 0.005 | R | R | R | 0.174± 0.013 |
